# Surveillance imaging of type II endoleak with contrast-enhanced ultrasound

**DOI:** 10.1259/bjrcr.20230053

**Published:** 2023-07-05

**Authors:** Filip Jelenak, Jaspreet Hira, Rachita Khot

**Affiliations:** 1 Des Moines University, Medicine & Health Sciences, IA, USA; 2 Department of Radiology and Medical Imaging, University of Virginia Medical Center/University of Virginia School of Medicine, Charlottesville, VA, USA

## Abstract

Type II endoleak is the most common type of endoleak after endovascular repair of abdominal aortic aneurysm and has been reported in up to 20–50% of patients. Patients undergo lifelong surveillance of aortic graft stents to monitor for endoleak. Contrast-enhanced ultrasound can be an adjunct to CT angiography (CTA) which is the preferred imaging modality for surveillance. However, CT angiography introduces challenges of recurring cost, exposure to ionizing radiation, and the need for iodinated contrast dye. We report a case using CEUS for the detection of type II endoleak.

## Introduction

An endoleak is a common complication after endovascular aneurysm repair (EVAR) and has been reported in up to 20–50% of patients.^1^ An endoleak is defined by persistent blood flow into the excluded aneurysm sac after EVAR. Endoleaks are divided into one of five variants based on the cause of this unwanted blood flow and are typically detected through surveillance CT angiography (CTA). Type II endoleak is the most common and usually occurs because of the presence of a patent inferior mesenteric or lumbar artery providing retrograde flow into the aneurysm sac.^1^ Patent intercostal arteries can lead to the same phenomenon in the thoracic aortic aneurysm.^1^ Given the risk of endoleak, patients routinely undergo annual surveillance imaging with CTA being the modality of choice.^2^ However, challenges are associated with repetitive CTA, including radiation exposure, use of iodinated contrast media, and increased cost.^2,3^ Alternative imaging options for surveillance that have been studied in the past include duplex ultrasound and MRI; however, they each have their shortfalls.^2,3^

Contrast-enhanced ultrasound (CEUS) is an ultrasound imaging technique that uses microbubble-based ultrasound contrast agents (UCAs). In recent years, CEUS has gained widespread acceptance as a valuable assessment tool for a wide range of medical conditions. CEUS has been studied in the evaluation of endoleak with several demonstrable advantages over traditional CTA. It is able to provide real-time dynamic information about the leak, its location, and flow characteristics that would not be available with the single time point acquisition of CTA.^2,4^ Additionally, CEUS has better sensitivity to low-flow endoleaks and is able to depict the excluded sac immediately adjacent to the stent graft, which is often obscured on CTA by metallic streak artifacts.^4^ Last, the typical CTA risks of contrast allergy, impaired renal function, and radiation load are avoided with CEUS.^4^

Currently, there has yet to be an agreement regarding the optimal diagnostic imaging modality for endoleak surveillance after EVAR.^2^ Herein, we present a case of a 70-year-old male patient who had EVAR for abdominal aortic aneurysm (AAA) and underwent surveillance imaging with CTA and subsequently CEUS to monitor the progression of Type II endoleak.

## Case presentation

A 70-year-old male patient initially presented to the vascular surgery clinic with a known diagnosis of a 4.5 cm infrarenal AAA noted on the screening examination. He was a former smoker with a 15-pack-year history. His family history was notable for ruptured aortic aneurysms resulting in his father’s death at age 63 years and his brother’s at age 50 years.

**Figure 1.**
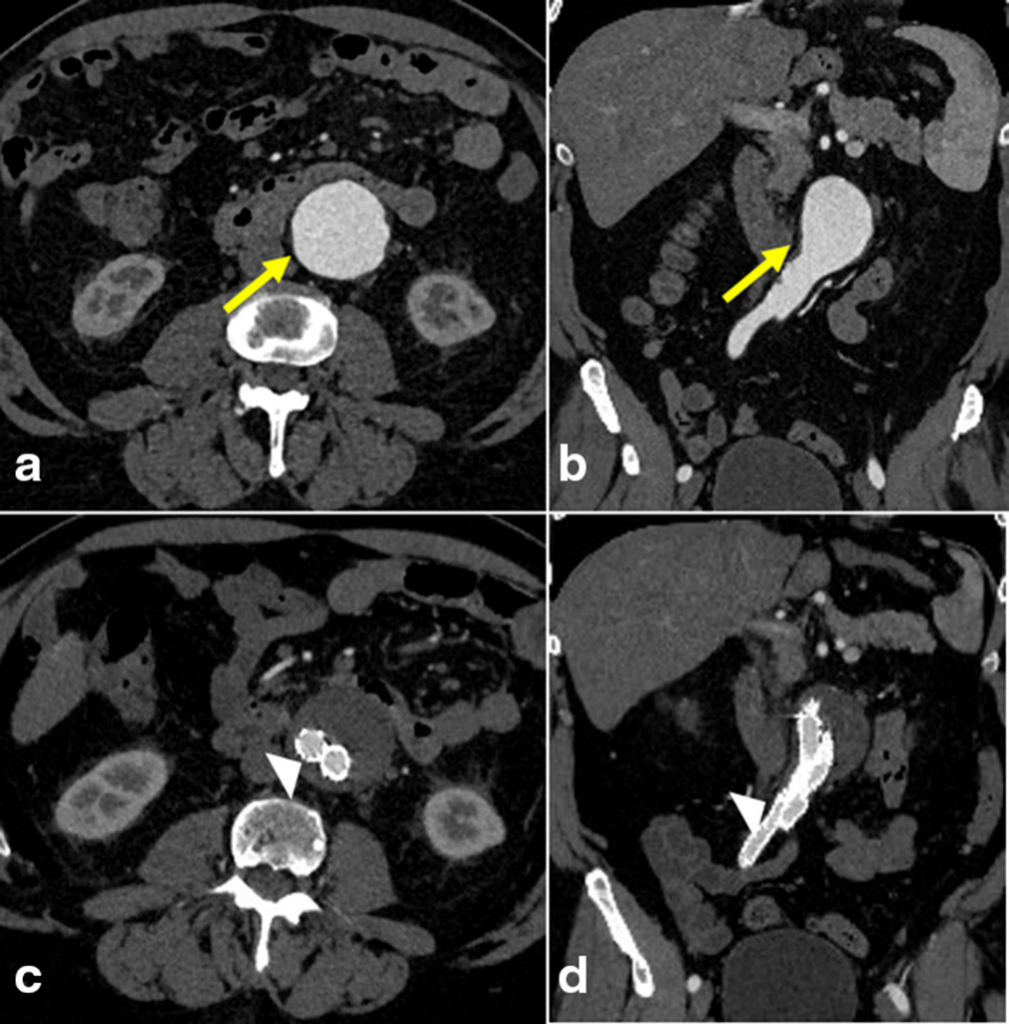
CT angiogram at the time of presentation. Axial (**a**) and coronal (**b**) images show a 4.7- × 4.5 cm infrarenal abdominal aortic aneurysm (arrow). Axial (**c**) and coronal (**d**) images show an aorto-bi-iliac endovascular stent graft (arrowhead). The aneurysm sac is stable in size. There is no contrast opacification of the excluded aneurysm sac on arterial or delayed venous phase images (latter not shown).

**Figure 2.**
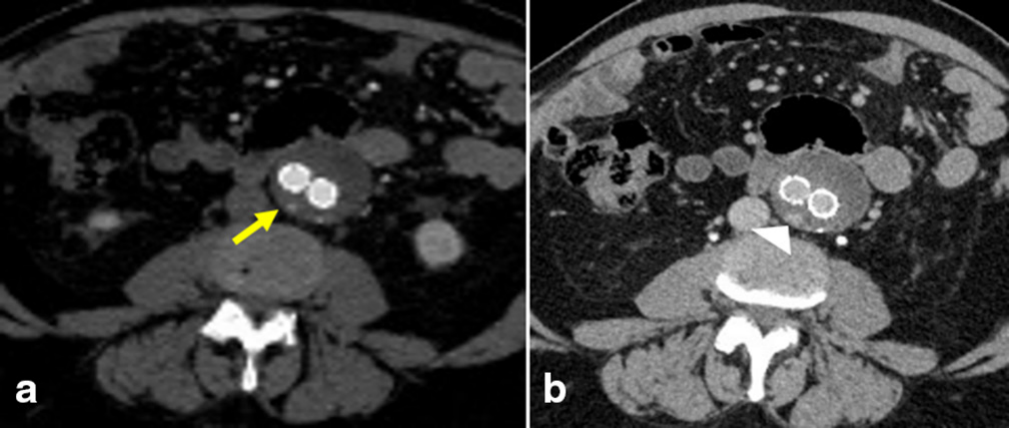
Axial arterial (**a**) and venous (**b**) phase post-contrast 1 year surveillance images status post-endovascular aneurysm repair show blush of arterial contrast (**a**, arrow) that progresses on venous phase (**b**, arrowhead) compatible with an endoleak. Given its posterior location and adjacent small lumbar vessels, this is likely a Type II endoleak arising from the L2 or L3 lumbar arteries.

**Figure 3.**
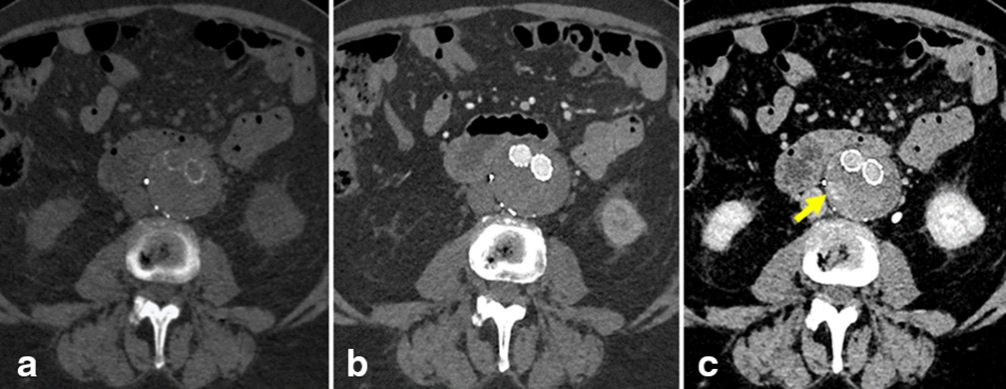
Axial virtual non-contrast (**a**) arterial (**b**) and venous (**c**) images at the level of the infrarenal abdominal aortic aneurysm status post-endovascular aortic aneurysm repair and coil embolization of the right L2 and L3 lumbar arteries. There is residual high-density material seen only on venous phase (c, arrow) within the aneurysm sac, which raises suspicion for residual slow-filling Type II endoleak.

At the initial presentation in 2017, the AAA measured 4.7 × 4.5 cm (Figure 1a and b). The patient proceeded to undergo repair of the infrarenal AAA with a bifurcated aorto-bi-iliac endovascular stent graft (Figure 1c and d). Subsequent imaging in 2019 showed a Type II endoleak arising from a right L2 or L3 lumbar artery with a stable aneurysm size (Figure 2). Following the stability of imaging findings in 2020, there was an increase in the aneurysm size in 2021, measuring 5.3 × 4.6 cm, likely secondary to the previously described endoleak. At this time, the patient underwent coil embolization of the endoleak arising from the collateral filling of a right L2 lumbar artery with prophylactic embolization of the right L3 lumbar artery with interventional radiology. Post-treatment imaging evaluation was limited secondary to extensive artifacts from the embolization material; however, a slow-filling Type II endoleak was still suspected (Figure 3).

**Figure 4.**
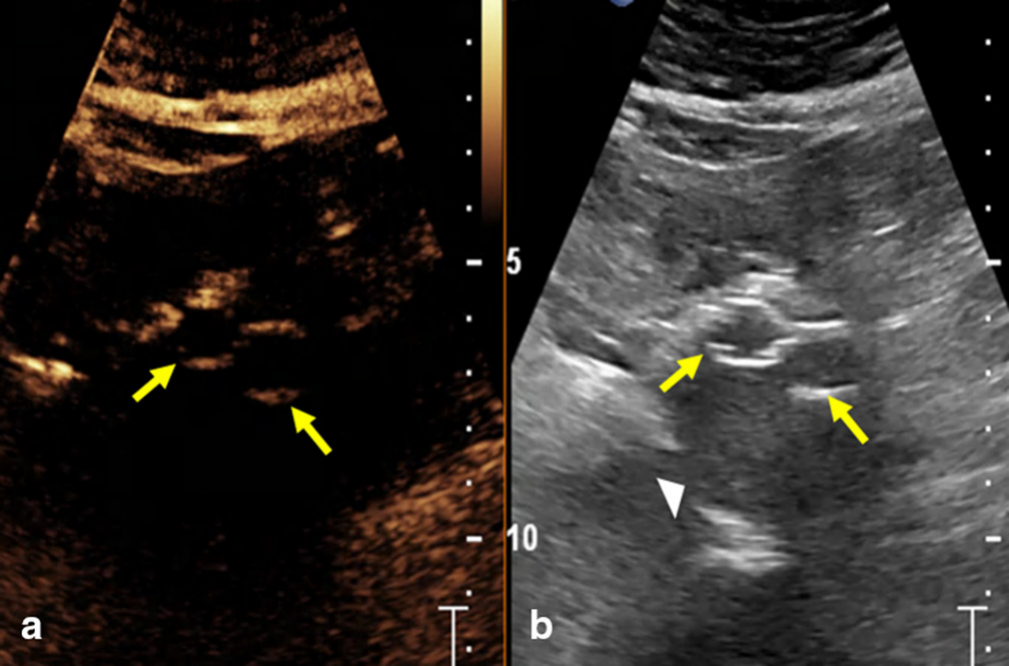
Transverse pre-contrast ultrasound image (**a**) and grayscale image (**b**) at the level of the infrarenal abdominal aortic aneurysm status post-endovascular aneurysm repair and coil embolization of the right L2 and L3 lumbar arteries. The echogenic iliac artery stent grafts (arrows) are readily visible on both ultrasound images. The aneurysm sac is anechoic and measures 5.1 cm (arrowhead).

**Figure 5.**
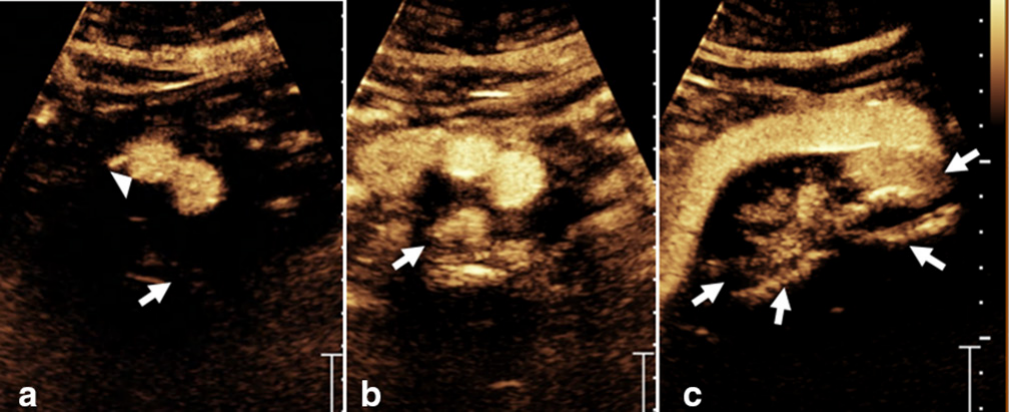
Transverse (**a, b**) and sagittal (**c**) post-contrast ultrasound images show a faint linear enhancing vessel outside the aneurysm sac (A, arrow) and contrast within the stent (**a**, arrowhead) obtained at 22 s after injection with progressively increased contrast in the aneurysm sac (**b**, arrow) with time. The filling of the aneurysmal sac and lumbar vessels are better seen on the sagittal image (**c**, arrows). The findings are compatible with persistent Type II endoleak.

Given the limitations of traditional CTA, the patient was selected to undergo CEUS to further evaluate the possible residual Type II endoleak. In 2022, CEUS demonstrated a persistent Type II endoleak (Figures 4 and 5).

## Discussion

CEUS uses intravenous injection of an UCA, which is composed of microbubbles of an inert gas surrounded by a stabilizing shell of phospholipid.^5^ This is subsequently observed with real-time ultrasound using a variety of non-linear imaging modes.^5^ CEUS allows for dynamic visualization and evaluation of the wash-in and wash-out phases of the UCA over several minutes.^6^ This can be applied to multiple aspects of radiology, including evaluation of focal solid organ lesions, urinary system, and indwelling catheters. This case describes its use in evaluating endoleak status post-EVAR.

Type II endoleak is readily identified on multiphase CTA by evaluating for a high-density contrast blush within the aneurysm sac on arterial phase imaging that progresses in size on venous phase images. Similar to CTA, CEUS shows increased echogenicity of the UCA within the aneurysm sac that progressively grows over the course of the ultrasound examination. CEUS can also be used in the assessment of different types of endoleak based on the timing and site of contrast arrival into the aneurysm sac.^7^ As demonstrated by Jawad et al,^7^ a Type I endoleak was identified because of early contrast filling of an aneurysm sac with simultaneous filling of the graft, characteristic of a Type I endoleak.^7^ As in our case, slow filling of the sac is compatible with a Type II endoleak on CEUS.^7^

Currently, the most commonly used modality for evaluation of endoleak after EVAR is CTA.^2^ Current guidelines recommend CT follow-up at 1 and 12 months during the first year post-operatively and additional CTA at 6 months if an abnormality is detected.^3,7^ However, this comes with increased risks of contrast-induced nephrotoxicity, radiation exposure, and recurring cost,^2^ which can be avoided by using UCA for surveillance imaging of endoleaks because UCAs are not nephrotoxic, have no radiation involved, and are cost-effective compared with CTA. UCAs are excreted through the lungs via exhalation.^8^ CEUS has shown a similar sensitivity and specificity to CT in detecting endoleaks.^2,7,9^ Gürtler et al^8^ reported a sensitivity of 97% and a specificity of 93% with CEUS in a study that followed 132 patients status post-EVAR. Cantisani et al^2^ also concluded that CEUS had a statistically significant higher sensitivity and specificity to color Doppler ultrasound, somewhat more accurate than CTA, and similar in accuracy to magnetic resonance angiography.

However, there are limitations to CEUS. As with all ultrasound examinations, patient body habitus is often a limiting factor, and individuals with larger body mass indexes are suboptimally assessed via CEUS. Real-time imaging usually requires the presence of a radiologist at the bedside during the administration of UCA to assess the adequacy of the images and need for repeat UCA administration. Although the overall cost to the patient is less than for CTA, a radiologist’s physical presence for CEUS and associated time loss demonstrate an inefficient allocation of resources. This can be overcome by training sonographers and radiologists to be available if needed. Aortic calcification within the aneurysm sac may mimic an endoleak, producing a false-positive result if not adequately identified on precontrast images.^7^ Lastly, ultrasound is an operator-dependent modality; therefore it cannot replace CTA, which remains a gold-standard for endoleak evaluation.

## Conclusion

Endoleak is a common complication after EVAR requiring lifelong imaging surveillance. CEUS is an safe imaging modality that not only reduces the overall cost but also has no adverse effect on renal function. Therefore, CEUS can be considered as an effective imaging adjunct to CTA for monitoring patients after EVAR.

## Learning points

- CEUS using microbubble-based ultrasound contrast agents has emerged as a valuable alternative to CTA for surveillance of endoleak evaluation.
- CEUS provides real-time dynamic information, and better sensitivity to low-flow endoleaks.
- CEUS offers advantages over CTA in surveillance imaging, including avoidance of risks associated with contrast allergies, impaired renal function, and radiation exposure.

Patient consent written informed consent was obtained from the patients for publication of this case report, including accompanying images.
